# Risk Prediction Model Development for Late On-Set Breast Cancer Screening in Low- and Middle-Income Societies: A Model Study for North Cyprus

**DOI:** 10.3390/healthcare8030213

**Published:** 2020-07-16

**Authors:** Ceasar Dubor Danladi, Nedime Serakinci

**Affiliations:** 1 Department of Medical Genetics, Institute of Health Sciences, Near East University, Nicosia 99138, Turkish Republic of North Cyprus; 20167382@std.neu.edu.tr; 2 Department of Medical Genetics, Faculty of Medicine, Near East University, Nicosia 99138, Turkish Republic of North Cyprus; 3 Department of Molecular Biology and Genetics, Faculty of Art and Sciences, Near East University, Nicosia 99138, Turkish Republic of North Cyprus

**Keywords:** breast cancer, mammogram, risk prediction model, North Cyprus

## Abstract

*Background*: Early detection of breast cancer alters the prognosis and tools that can predict the risk for breast cancer in women will have a significant impact on healthcare systems in low- and middle-income regions, such as North Cyprus. *Objective*: In this study, we developed a simple breast cancer risk model for the women of North Cyprus. *Methods*: Data from 655 women, consisting of 318 breast cancer cases and 337 hospital-based controls, was used to develop and internally validate the model, external validation was carried out using, 653 women consisting of 126 cases and 527 controls. Data were obtained from medical records and interviews after informed consent. *Results*: A model was derived that consisted of age ≥50 years and <50 years and the presence and absence of >1 first-degree relatives (FDR) with breast cancer. From internal and external validations the model’s AUCs were, 0.66 (95% CI = 0.62–0.70) and 0.69 (95% CI = 0.63–0.74) respectively. *Conclusions*: A unique model for risk prediction of breast cancer was developed to aid in identifying high-risk women from North Cyprus that can benefit from mammogram screening. Further study on a large scale that includes environmental risk factors is warranted.

## 1. Introduction

Breast cancer is a disease that has remained the leading cause of death in women worldwide [1]. In 2018, over two million women were diagnosed with breast cancer in 140 of 184 nations [2]. Breast cancer incidence and deaths occur mostly in low- and middle-income countries [3,4]. One-half of all breast cancer cases exist in low- and middle-income societies, with 62% of the world’s breast cancer deaths [5], whereas in the United States, approximately 268,600 new cases of breast cancer are diagnosed each year, and the mortality rate is decreasing [6], with 89.7% five-year survival rate [1,7]. These portray inequality in global health standards.

Additionally, the prognosis is always poor for women living in low- and middle-income countries [7,8], because of lack of risk forecast, late detection, late intervention, environmental factors and lack of education on routine care.

Northern Cyprus, can be regarded as a middle-income society, small, enclosed, ideal for epidemiological research, has a typical western Mediterranean lifestyle, with living conditions and diets that should be favorable for good health [9,10]. Previously, Hincal et al. investigated the prevalence of cancer in Northern Cyprus, compared to different European countries between 1990 and 2004, and showed that breast cancer was the most common cancer in women and diagnosed at a lower average age than Northern and Southern Europe [11]. Later on, Pervaiz et al. also found that out of 1395 enlisted cancer cases between 2007 and 2012, 665 (47.67%) were women, and breast cancer was the most common cancer type among the women [12]. The lifespan of women in the developing world is increasing, and thus many more women are reaching an age where breast cancer rates are high [13].

In high-income countries, the incidence and mortality associated with breast cancer are decreasing. These may be accounted for because of access to mammogram screening, effective health services, and good execution of breast cancer prevention campaigns, as well as primary prevention policies and guidelines [14,15,16]. Yet the burden on healthcare systems is increasing in low- and middle-income countries.

Despite the fact, mammogram screening has been supported for the early detection of breast cancer, it is not cost-effective and feasible for developing countries, because of the large population of women, underfunding, infrastructure and human expertise needed [16,17,18,19,20,21,22,23,24]. Therefore, such countries can benefit from cost-effective and efficient risk assessment screening strategies, which do not depend on tertiary or specialized healthcare. Denny et al. explored some cost-effective methods that can be used to reduce the gap between developing countries and developed countries for breast cancer early detection, prevention, and care [25].

Risk prediction models can be used to identify high-risk women that will be eligible for mammogram screening. Thus, reducing the overload on the limited facilities available and narrowing the focus on the appropriate group can also reduce administering unnecessary radiation to women, who are not eligible, while at the same time reducing the economic burden on the government. Currently, several comprehensive breast cancer risk assessment tools exist that incorporate various risk factors for the calculation of breast cancer risk [1].

Many risk factors play a role in the development of breast cancer. Environmental factors that contribute to interethnic variations in risk, age, lifestyle factors, endogenous and exogenous hormonal exposure, childbearing, breastfeeding, reduced physical activity, dietary consumption, certain reproductive factors (low parity, late age at first pregnancy), body size/obesity contribute to differences in incidence [26], family history and genetic influence are particularly strong risk factors for breast cancer [27,28] and breast density, which is also a predictor for breast cancer [29].

Breast cancer risk assessment models are categorized into empirical models or genetic models [30]. A pattern of genetic inheritance of breast cancer risk is considered by the genetic models. Several risk assessment models have been proposed [30,31,32]. The Breast and Ovarian Analysis of Disease Incidence and Carrier Estimation Algorithm (BOADICEA) model includes a polygenic component, which allows for the familial correlation that is not captured by mutations in Breast Cancer genes (BRCA) 1 or 2 [33], while the International Breast Cancer Intervention Study (IBIS) model, accommodates such residual familial correlation by incorporating a latent common autosomal dominant low-risk gene [34]. The National Cancer Institute’s Breast Cancer Risk Assessment Tool (BCRAT) also known as the “Gail Model” [35], estimates risk by incorporating the familial influence of first-degree relatives with breast cancer. The models are known for better performances in predicting high-risk women in the regions, which they were developed [36]. The development of a breast cancer risk model for the women of North Cyprus will allow for the early identification of high-risk women that will lead to early preventive interventions [37] and will save lives.

## 2. Materials and Methods

This study was carried out in the hospital, Burhan Nalbantoglu Devlet Hastanesi, Lefkosa, North Cyprus. This hospital treats all breast cancer cases in North Cyprus. Ethical approvals were obtained from Near East University, scientific research evaluation ethics committee, and Burhan Nalbantoglu Devlet Hastanesi’s ethics committee, before the research was carried out (YDU/2018/55-523). All methods were performed following the relevant guidelines and regulations.

### 2.1. Study Population

The retrospective dataset of 655 women, collected from April 2018 to December 2018, was used to derive the model. A total of 318 women had newly confirmed breast cancer, and 337 women were without breast cancer. Women with a history of lobular or ductal carcinoma in-situ were excluded. Only participants between the ages of 30 to 84 years were included in the whole study groups. Informed consent to participate was obtained after the aims of the study were explained by a medical professional.

### 2.2. Data Collection:

Retrospective medical and demographic information of all participants were collected through interviews.

The interview included: age, age at menarche, age at first delivery, menopausal status, presence or absence of benign breast disease, history of breast cancer in first-degree relatives or other relatives, history of hormone replacement therapy including estrogen/progestin and breast density.

Sampling size was based on the following calculations:

(1) $$\;n=\frac{N\times t^{2}p\times q}{\left(N-1\right)d^{2}+t^{2}\times p\times q}$$

*N* = 121,257 (Women Population Size);

*t* = *t* value = 1.96, (at α = 0.05);

*p* = (prevalence rate) = 91/100000 = 0.00091 (Expected Frequency);

*q* = 1 − *p* = 0.99909;

*d* = (Acceptable margin of Error) = 0.001;

The required sample size = 317.8 women.

### 2.3. Statistical Analysis

The frequency of the risk factors of the study group was analyzed using descriptive statistics. An initial multivariable logistic regression was carried out. The significant variables were considered for further multivariable logistic regression. A forward multivariable logistic regression was used to access the final model. In the multivariable regression analysis, the categories that conferred protection against breast cancer were used as the reference. All statistical analysis was performed with IBM Spss (IBM, Armonk, NY, USA).

#### 2.3.1. Internal Validation

The whole dataset of 655 women, consisting of 318 breast cancer cases and 337 without breast cancer from the derived phase, was used to internally validate the model using bootstrap with 200 repetitions [38,39]. For each bootstrap, the derived model was fitted and the risk of breast cancer was estimated. The correlation between the observed and predicted values of breast cancer was estimated in the bootstrap data (called Dboot) and derived data (called Doriginal) using the Somer’D coefficient [38]. The optimism bias was assessed by subtracting Doriginal from Dboot.

#### 2.3.2. External Validation

Separate information of 653 women, consisting of 126 cancer cases and 527 women without breast cancer, collected between November 2018 to January 2020, were used to externally validate the model. Total scores for individuals were calculated based on the derived scoring scheme, and the c-statistic was then estimated.

## 3. Results

A total of 655 women were used to derive the model. Among them, 51.1% were above 50 years; 48.5% (318) of the women had breast cancer, while the rest reported with no breast cancer. A > 1 FDR with breast cancer was reported in 9.9% of the study population (Table 1).

A total of 10 variables were analyzed to access the risk model, After an initial logistic regression of all the variables and two successive forward multivariable logistic regression, the risk factors that were observed to be insignificant were eliminated at each step, then we arrived at two significant risk predictors that comprised the final model, that is >1 FDR with breast cancer OR = 3.0 (95% CI 1.6–5.4) and age above 50 years OR = 3.0 (95% CI 2.2–4.1). The c-statistic of the final model on internal validation was 0.66 (95% CI 0.62–0.70). The estimated coefficients of the two variables served as the basis for the scoring with a range of 0–2. The risk scores were stratified into three groups; low-risk (0) women <50 years and with no >1 FDR with breast cancer, moderate-risk (1) women with >1 FDR with breast cancer or ≥50 years and high-risk (2)—that is, women—≥50 years and with >1 FDR with breast cancer.

From the internal validation the average Doriginal and Dboot were (0.328) and (0.350) respectively. The bias or optimism was (0.022).

**Figure 1:**
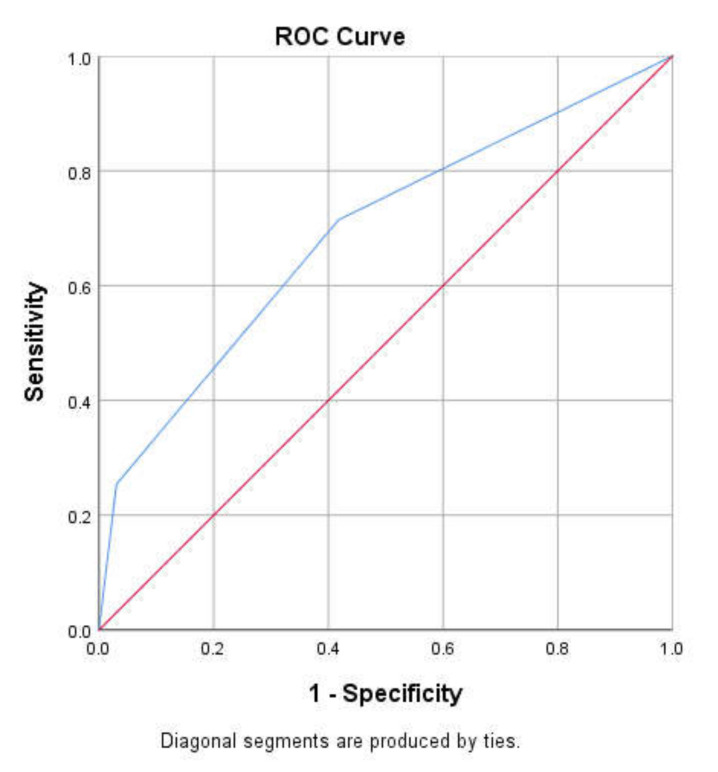
The receiver operating characteristics (ROC) curve of the simple model. This shows the discriminatory accuracy of the model. A value of 1 indicates perfect discrimination while 0.5 is by chance going to discriminate which woman will or will not have breast cancer. The red diagonal line represents the reference point. The blue line is the ROC curve of the simple model.

Separate information from 653 women, was used for external validation, consisting of 126 women with breast cancer and 527 women without breast cancer. From Figure 1, the c-statistics was 0.69 (95% CI 0.63–0.74), and the sensitivity and specificity are as shown in Table 2.

## 4. Discussions

In comparison, to a recent validation study carried out on a large cohort, the model showed similar c-statistics, sensitivity, and specificity to the Gail, IBIS and BOADICEA models [40]. The other models were developed for populations with base-line etiological risk factors that differed from our setting [36]. So, the model was developed to include only the base-line risk factors that are peculiar to the women of North Cyprus at the moment. Yet, the model can aid in categorizing high-risk women. The model utilized age ≥50 years and <50 years and presence or absence of >1 FDR with breast cancer in determining the risk of breast cancer.

Inherited factors elucidate just about a quarter of breast cancer risk [41]. Meta and pooled studies have demonstrated that breast cancer risk is around twice higher in women with one FDR with breast cancer than women with no FDR with breast cancer. The risk increases with a large number of affected FDR or other relatives affected under 50 years [42,43,44]. BRCA 1 and 2 mutations explain the molecular pathogenesis behind 15–20% of cases with FDR with breast cancer [41,45] and about 80–85% are as a result of genetic mutations that occur due to the aging process and lifestyle-related risk factors [42,46,47,48,49,50,51,52,53,54,55,56].

Aging plays a role in the pathogenesis of breast cancer because of genetic instability, telomere attrition, epigenetic alteration, stem cell exhaustion associated with aging.

From 50 years to 70 years and above, the risk of breast cancer increases [57].

The assessment of risk is vital for the management of breast cancer. High-risk women will not automatically have breast cancer, but they are strongly advised to visit cancer clinics. They should have a mammogram at least yearly, for this has been shown to increase detection rates and reduce mortality [58,59,60,61,62].

We recommend that the women of North Cyprus categorized as high-risk for breast cancer can benefit from regular monitoring using mammograms, early detection, and preventive interventions such as healthy lifestyle and medication.

Those women with moderate-risk of breast cancer, should undergo screening of breast cancer risk using mammograms biennially. For women above 50 years with no >1 FDR with breast cancer, while those women below 50 years with >1 FDR with breast cancer can consider the use of medications, such as tamoxifen or raloxifene to reduce the risk of developing breast cancer. Risk and benefits have to be assessed by a medical professional.

Low-risk women are required to indulge in primary care since their risk is not different from the general population, but this status is liable to change in the presence of modifiable risk factors. Thus, these women are encouraged to maintain a healthy lifestyle and breast care. Increased awareness of existing and identified risk factors will aid in evaluating their current status and make the appropriate decision for mammogram screening if warranted. These recommendations are summarized in Table 3.

This model can serve as a simple, noninvasive, alternative screening for the identification of high-risk women, thus streamlining the focus of the limited mammogram resources to the right group in low- and middle-income countries such as North Cyprus. Using this model will also reduce unnecessary mammograms needed and radiation exposure to potentially low-risk women. The use of the risk prediction model has additional advantages, as it is not dependent on physical examination, easy to utilize and implement, being cost-effective and will enhance outcome and survival for women categorized as high-risk.

The poor funding of health systems in low- and middle-income countries causes problems in the implementation of mammogram screening programs, thereby leaving many of the women out and only a few from the urban centers with insurance policies are privileged to participate.

This model can serve as additional screening tools that will aid in identifying women at high-risk that will need an immediate mammogram, thus reducing the burden on the already constrained facilities and hence saving lives.

The progress in mobile internet services in low- and middle-income countries has made online materials easily accessible to everyone. Educating and empowering women on how to use the risk prediction tools online will drastically reduce the number of high-risk women that cannot have access to early detection facilities. These will protect more women at the individual and population levels.

This initiative is maybe similar to the mobile health (mhealth) initiative launched by the world health organization in 2012, whereby mobile phones were used to improve the prevention, detection, and management of diseases in low-income countries [63]. The risk assessment model can be incorporated into the mhealth features, thereby empowering women. Low-risk women through mhealth, can benefit from primary care, where healthcare teams deliver healthcare remotely, through audio, video and text.

Risk prediction tools at the moment can serve as cost-saving tools. However, the benefits can only be maximized when all identified high-risk women can receive further confirmation screening and treatment. Therefore, identified high-risk women will still need to visit healthcare centers for counseling and prophylactic treatment.

While the models intend to ascertain the risk for an individual, the risk factors utilized to depend on population risk from epidemiological investigations. Therefore, more studies have to be carried out among various populations of women in other to identify new lifestyle/environmental factors, biomarkers, genetic markers and incidence rates that are peculiar to that group, which can be incorporated into prospective risk models because the possibility of identifying those at high-risk would be enhanced by using a comprehensive risk model that integrates all known risk factors [64]. Because our enrollment of breast cancer patients for this investigation is not specifically aimed to gather the early on-set breast cancer patients, we assume that our model is more suitable for the late on-set breast cancer risk prediction. That is why we added the phrase “Late On-Set Breast Cancer Screening” into the manuscript’s title. We think that the early on-set breast cancer risk prediction model should be developed in the future by using a specific cohort made up by enrollment of early on-set breast cancer patients.

The weakness of our study is the fact that it was based on retrospectively collected data. However, the collecting process was done independently, so unlikely to have altered the results and caused bias. The AUC estimates are bound to be biased since our validation was carried out on a case-control group, but this was minimized [64]. The internal and external validation was done using data from the same hospital but collected at different times.

Information on environmental risk factors was not collected, and this may have created a gap in the risk factors of the studied population.

Despite these biases and limitations, the urgent need for a risk prediction model in providing relevant breast cancer control in developing societies such as North Cyprus outweighs the shortfalls.

## 5. Conclusions

Our results demonstrated that this newly developed breast cancer risk prediction model is a simple, cost-effective, and noninvasive tool for the identification of high-risk women in North Cyprus that can be eligible for a mammogram. It may serve as a gatekeeper for a mammogram and a radiation saving tool for low-risk women, by reducing unnecessary mammograms and thereby decreasing health costs. This model is suitable for the prediction of late on-set breast cancer risk and further studies on a larger study group, including environmental risk factors will be needed to improve the model.

## Figures and Tables

**Table 1:** The frequencies and percentages of variables in the datasets used for the model development.

Characteristics	Frequencies (%)
Reproductive History	
Age at menarche	
≥14 years	155 (23.7%)
14–13 years	421 (64.3%)
>12–<13 years	0 (0%)
<12 years	79 (12.1%)
Age at first birth	
≥30 years	58 (8.9%)
25–29 years	142 (21.7%)
20–24 years	238 (36.3%)
<20 years	167 (25.5%)
Nulliparous	50 (7.8%)
Menopausal status	
Premenopausal	314 (47.9%)
Perimenopausal	13 (2.0%)
Postmenopausal	328 (50.1%)
Breastfeeding	
≥24 months	285 (43.5%)
<24–>18 months	0 (0%)
18–12 months	236 (36.0%)
<12–>6 months	0 (0%)
<6 months	83 (12.7%)
Never	51 (7.8%)
Breast density	
Extremely dense	58 (8.9%)
Heterogeneously dense	334 (51.0%)
Almost entirely fatty	263 (40.2%)
Demographic Data	
>1 First degree relatives	
Yes	65 (9.9%)
No	590 (90.1%)
Second degree relatives	
Yes	41 (6.3%)
No	614 (93.7%)
Hormone Replacement Therapy	
Yes	17 (2.6%)
No	638 (97.4%)
Breast biopsy	
Yes	85 (13.0%)
No	570 (87.0%)
Age	
>50	320 (48.9%)
≤50	335 (51.1%)
Disease status	
Breast cancer cases	318 (48.5%)
Without breast cancer	337 (51.5%)

**Table 2:** Sensitivity and specificity of the simple model on external validation.

Cut-Off Value	Sensitivity	Specificity
0.5–1.0	71.4%	41.7%

**Table 3:** Summarized recommended guidelines for the management of breast cancer risk screened by the simple model in the women of North Cyprus.

Risk Status	Suggestions/Advice	Outcome
High-risk (>1 FDR and ≥50 years)	Regular monitoring, early detection and preventive interventions	Reduced mortality
Moderate-risk (>1 FDR or ≥50 years)	Further screening to access risk	Prevention of occurrence
Low-risk (No FDR, <50 years)	Primary care	Awareness and prevention
